# Supplementary figures and images for: Brain transcriptomes of harbor seals demonstrate gene expression patterns of animals undergoing a metabolic disease and a viral infection
Source: PeerJ. 2016 Dec 22;4:e2819. doi: 10.7717/peerj.2819 (PMC5182994; doi:10.7717/peerj.2819)

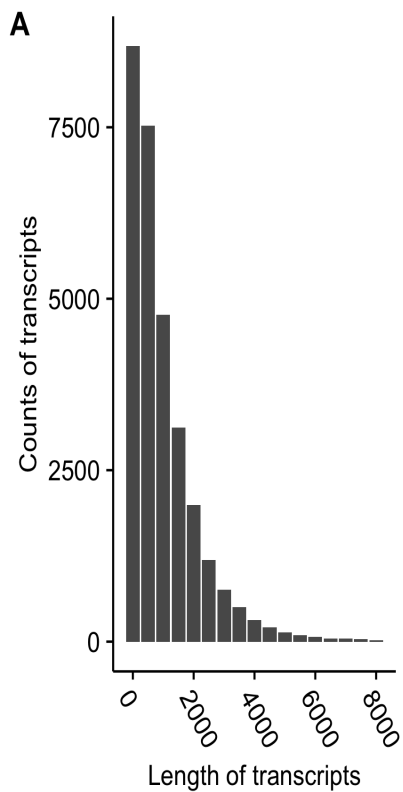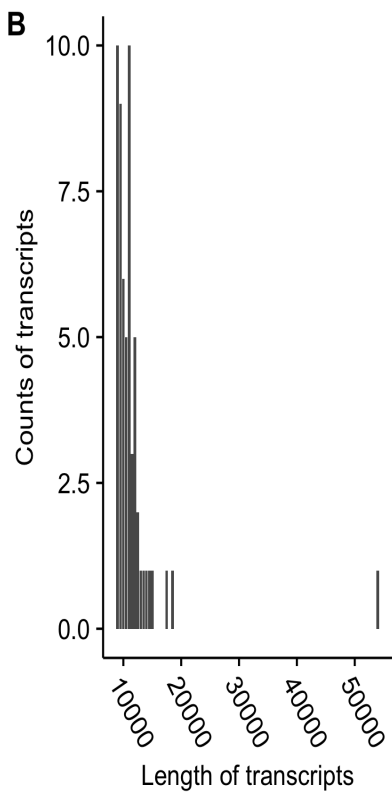

Supplement: Figure S1 — (A) The length and count distribution of the harbor seal brain transcriptome with transcript lengths < 8,500 and (B) the length and count distribution of the harbor seal brain transcriptome with transcript lengths > 8,500. [file peerj-04-2819-s001.pdf]

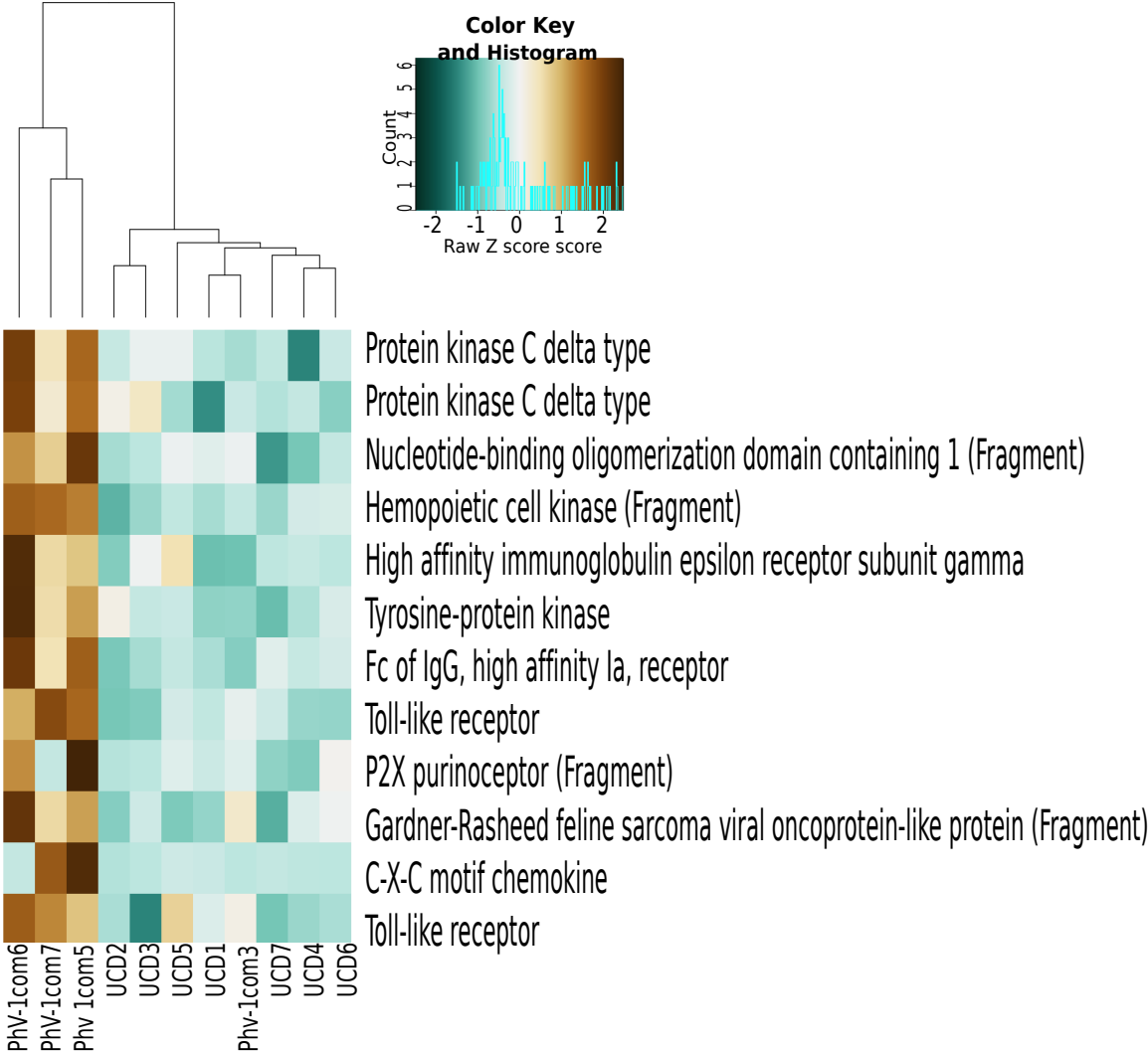

Supplement: Figure S2 — Heatmap hierarchical clustering of normalized gene counts expressed in rlog transformation (row z-score) from PhV1com and UCD harbor seals. [file peerj-04-2819-s002.pdf]
