# Supplementary material for: Brain transcriptomes of harbor seals demonstrate gene expression patterns of animals undergoing a metabolic disease and a viral infection
Source: PeerJ. 2016 Dec 22;4:e2819. doi: 10.7717/peerj.2819 (PMC5182994; doi:10.7717/peerj.2819)
Supplement: Table S1 [file peerj-04-2819-s003.docx]

| Method for transcriptome assembly | #transcripts | # of Open reading frames (ORFs) | % of Open reading frames (ORFs) | N50 | Shortest transcript | Longest  transcript | % of sequences that aligned |
| --- | --- | --- | --- | --- | --- | --- | --- |
| *De novo* only with trinity | 874,457 | 57,557 | 6.5 | 804 | 224 | 29,623 | Not applicable |
| Weddell seal genome guided and *de novo with* trinity | 1,383,525 | 67,643 | 4.9 | 1,135 | 224 | 27,818 | 76.51% |
| Weddell seal transcriptome guided and *de novo* with trinity | 32,856 | 21,646 | 65.9 | 1,994 | 224 | 54,385 | 27.43% |
